# P21-dependent G _1_ arrest with downregulation of cyclin D1 and upregulation of cyclin E by the histone deacetylase inhibitor FR901228

**DOI:** 10.1054/bjoc.2000.1327

**Published:** 2000-08-17

**Authors:** V Sandor, A Senderowicz, S Mertins, D Sackett, E Sausville, M V Blagosklonny, S E Bates

**Affiliations:** Medicine Branch, DSC, NCI, NIH, Bethesda, MD, 20892; Developmental Therapeutics Program, NCI, NIH, Bethesda, MD, 20892, USA

**Keywords:** experimental therapeutic, cell cycle, cyclin, p21, cytotoxicity

## Abstract

Depsipeptide, FR901228, a novel cyclic peptide inhibitor of histone deacetylase with a unique cytotoxicity profile is currently in phase I clinical trials. Here we demonstrate that, in addition to G2/M arrest, FR901228 causes G1 arrest with Rb hypophosphorylation. In vitro kinase assays demonstrated no direct inhibition of CDK activity, however, an inhibition was observed in CDKs extracted from cells exposed to FR901228. Cyclin D1 protein disappeared between 6 and 12 hours after treatment with FR901228, whereas cyclin E was upregulated. While it did not induce wt p53, FR901228 did induce p21^WAF1/CIP1^in a p53-independent manner. Cell clones lacking p21 were not arrested in G1 phase, but continued DNA synthesis and were arrested in G2/M phase following FR901228 treatment. Finally, FR901228 blunted ERK-2/MAPK activation by EGF whereas early signal transduction events remained intact since overall cellular tyrosine phosphorylation after EGF stimulation was unaffected. Thus, FR901228, while not directly inhibiting kinase activity, causes cyclin D1 downregulation and a p53-independent p21 induction, leading to inhibition of CDK and dephosphorylation of Rb resulting in growth arrest in the early G1 phase. In contrast to the G1 arrest, the G2/M arrest is p21-independent, but is associated with significant cytotoxicity. © 2000 Cancer Research Campaign


*British Journal of Cancer* (2000) **83**(6), 817–825
© 2000 Cancer Research Campaign
doi: 10.1054/ bjoc.2000.1327, available online at http://www.idealibrary.com on IDEAL®

### P21-dependent $G_1$ arrest with downregulation of cyclin D1 and upregulation of cyclin E by the histone deacetylase inhibitor FR901228

**V Sandor[1], A Senderowicz[2], S Mertins[1], D Sackett[1], E Sausville[2], MV Blagosklonny[1] and SE Bates[1]**

[1]Medicine Branch, DSC, NCI, NIH, Bethesda, MD 20892; [2]Developmental Therapeutics Program, NCI, NIH, Bethesda, MD 20892, USA

**Summary** Depsipeptide, FR901228, a novel cyclic peptide inhibitor of histone deacetylase with a unique cytotoxicity profile is currently in phase I clinical trials. Here we demonstrate that, in addition to G2/M arrest, FR901228 causes G1 arrest with Rb hypophosphorylation. In vitro kinase assays demonstrated no direct inhibition of CDK activity, however, an inhibition was observed in CDKs extracted from cells exposed to FR901228. Cyclin D1 protein disappeared between 6 and 12 hours after treatment with FR901228, whereas cyclin E was upregulated. While it did not induce wt p53, FR901228 did induce $p21^{WAF1/CIP1}$ in a p53-independent manner. Cell clones lacking p21 were not arrested in G1 phase, but continued DNA synthesis and were arrested in G2/M phase following FR901228 treatment. Finally, FR901228 blunted ERK-2/MAPK activation by EGF whereas early signal transduction events remained intact since overall cellular tyrosine phosphorylation after EGF stimulation was unaffected. Thus, FR901228, while not directly inhibiting kinase activity, causes cyclin D1 downregulation and a p53-independent p21 induction, leading to inhibition of CDK and dephosphorylation of Rb resulting in growth arrest in the early G1 phase. In contrast to the G1 arrest, the G2/M arrest is p21-independent, but is associated with significant cytotoxicity. © 2000 Cancer Research Campaign

**Keywords:** experimental therapeutic; cell cycle; cyclin; p21; cytotoxicity

Current classes of chemotherapeutic agents offer limited therapeutic benefit for many human malignancies. New classes of agents with diverse structures and molecular targets are, therefore, of interest in the pursuit of improved anticancer agents. While empirical evaluation of the clinical efficacy of such new compounds still forms the cornerstone of their development as therapeutic agents, an understanding of their molecular mechanism of action is important to developing rational strategies for clinical evaluation.

FR901228 (NSC 630176), is a cytotoxic depsipeptide isolated by the Fujisawa Pharmaceutical Company from the fermentation broth of *Chromobacterium violaceum* (Ueda et al, 1994b). Initial characterization of the compound showed it to be cytotoxic at nanomolar concentrations in several in vitro and in vivo models (Ueda et al, 1994a,b). FR901228 was found to downregulate c-myc mRNA and to cause a G1 cell cycle arrest in H-ras transformed NIH 3T3 cells (Ueda et al, 1994c) and also to downregulate c-myc in activated T lymphocytes (Wang et al, 1998). The products of several oncogenes have been identified as components of signal transduction pathways, or of autocrine loops involving signal transduction pathways (Hunter, 1997). For example, the oncogenes ErbB2, Ras and Raf are components of the mitogen activated protein kinase (MAP kinase) pathway that have been subverted by mutation or overexpression, provoking uncontrolled signalling. Activation of the MAPK pathway induces cyclin D1 (Aktas et al, 1997; Zou et al, 1997) which in turn activates CDK causing Rb phosphorylation, determining the restriction point in moving from G1 into S phase (Weinberg, 1995). Phosphorylation of Rb causes release of the E2F transcription factor (Nevins, 1998). This transcriptionally induces c-myc and other genes that are required for initiation of DNA synthesis (Johnson et al, 1993; Oswald et al, 1994; Weinberg, 1995). In contrast, downregulation of cyclin D1 or upregulation of CDK inhibitors such as p21 causes Rb dephosphorylation and growth arrest (Sherr, 1999).

Histone acetylation, originally discovered in 1964, provides an enzymatic mechanism to regulate transcription by affecting the interaction between DNA and histones. Recently, it has been determined that dephosphorylated Rb bound to E2F associates with a histone deacetylase to effect transcriptional repression (Brehm et al, 1998; Luo et al, 1998; Magnaghi-Jaulin et al, 1998). Inhibitors of histone deacetylase, although counterintuitive, have the capacity to inhibit cell growth while increasing gene expression. Several histone deacetylase (HDAC) inhibitors were previously studied as differentiating agents (Candido et al, 1978; Richon et al, 1998). For example, HDAC inhibitors synergize with retinoic acid to stimulate leukaemia cell differentiation (Grignani et al, 1998; Lin et al, 1998; Kosugi et al, 1999). It has been demonstrated that FR901228 is a novel histone deacetylase inhibitor (Nakajima et al, 1998). Like other inhibitors (Sowa et al, 1997), FR901228 induces p21 in a p53-independent manner (Rajgolikar et al, 1998). However, the significance of this induction in the mechanism of growth arrest or cytotoxicity by FR901228 is unclear.

To further characterize the mechanism of action of FR901228, given its ability to cause G1 and G2/M cell cycle arrest, we investigated the effect of FR901228 on the regulatory mechanisms involved in the G1-to-S transition. We report that FR901228

*Received 14 February 2000*
*Revised 27 April 2000*
*Accepted 1 May 2000*

*Correspondence to:* SE Bates downregulated cyclin D1 and upregulated CDK inhibitor p21$^{WAF1/CIP1}$ resulting in inhibition of CDK activity and Rb dephosphorylation and G1 arrest. Cells lacking p21 did not undergo G1 arrest, continued DNA synthesis and were arrested in the G2/M phase of the cell cycle.

### METHODS

### Cell lines

MCF-10A is an EGF-dependent immortalized but non-transformed breast cell line derived from human fibrocystic mammary tissue (Soule et al, 1990). MCF-10A cells were maintained in DMEM/F12 medium (Biofluids, Rockville, MD, USA) with 5% horse serum (Gibco BRL, Rockville, MD, USA), 10 ng/ml human EGF (Upstate Biotechnology, Lake Placid, NY, USA), 500 ng/ml hydrocortisone (Sigma, St Louis, MO, USA), and 100 ng/ml bovine insulin (Sigma, St Louis, MO, USA). All other cell lines, PC-3 (prostate carcinoma), SW620 (colon carcinoma), IGROV (ovarian carcinoma), DU145 (prostate carcinoma), MCF-7 (breast carcinoma), and A549 (lung carcinoma) were obtained from the NCI drug screen and maintained as described previously (Wosikowski et al, 1996). For p21 studies, the human colon carcinoma cell line HCT116 and two clones lacking p21 were used. The clones, S4 and S14 (both p21–/–), were a gift from Dr B Vogelstein (Johns Hopkins University) and described previously (Waldman et al, 1996; Blagosklonny et al, 1997; Bunz et al, 1998). Ad-p21, a p21 expressing adenovirus, was obtained from Dr WS El-Deiry (University of Pennsylvania, Philadelphia) and the viral titre was determined as previously described (Blagosklonny et al, 1997). Multiplicity of infection (MOI) is defined as the ratio of total number of viruses used in a particular infection per number of cancer cells to be infected (i.e., number of viruses per cell).

### Cell cycle analysis

Cells were harvested by trypsinization while in the exponential growth phase (50–70% confluence). Cells were washed twice with PBS and resuspended in 75% ethanol in PBS and kept at 4°C for at least 30 minutes. Prior to analysis, cells were washed again with PBS and resuspended and incubated for 30 min in propidium iodide staining solution containing 0.05 mg/ml propidium iodide (Sigma, St Louis, MO, USA), 1 mM EDTA, 0.1% Triton-X-100 and 1 mg/ml RNAse A in PBS. The suspension was then passed through a nylon mesh filter and analysed on a Becton Dickinson FACScan.

### Immunoblotting

Cells in 10 cm dishes were scraped into cell lysis buffer containing 20 mM Tris, 150 mM NaCl, 2 mM EDTA, 0.5% NP-40, 0.5% deoxycholate, and 0.1% SDS, with phosphatase inhibitors (1 mM sodium orthovanadate and 25 mM NaF) and protease inhibitors (PMSF, leupeptin and aprotonin). Lysates were clarified by spinning at 14 000 rpm for 15 minutes and protein concentrations determined using the BioRad colorimetric assay. Proteins were then separated by standard SDS-PAGE chromatography and transferred to PVDF (Millipore) membranes by electroblotting in 10 mM CAPS (Sigma, St Louis, MO, USA) buffer at pH 11.0. Immunoblotting was performed using primary antibodies at the following dilutions in 1% milk or 4% BSA (for anti-tyrosine phosphate anti-activated EGFR) in Tris buffered saline (150 mM NaCl, 20 mM Tris-HCl pH = 7.5) with 0.05% Tween 20: anti-ERK2, anti-RB, anti-p53, anti p21WAF1, anti-cyclin D1, anti-cyclin E (Santa Cruz Biotech, Santa Cruz, CA, USA) at 1:100, anti-tyrosine phosphate, (Signal Transduction Laboratories, Lexington, KY, USA) at 1:1000, anti-cyclin E (Pharmigen, San Diego, CA, USA) at 1:1000. Immunoblots were developed using an HRP-conjugated secondary antibody (Bio Rad) and a chemiluminescence detection kit (Dupont NEN, Wilminghton, DE, USA).

### Immune complex kinase assays

Cells were lysed with buffer containing 50 mM Hepes (pH 7.5), 20 mM EDTA, 0.5% NP 40, 1 mM ABESF, 5 mg/ml aprotinin, 5 mg/ml leupeptin, 10 mM β-glycerophosphate, 0.5 mM NaF and 0.4 mM NaVO4. The lysate was centrifuged at 15 000 ***g*** for 30 min at 4°C. The supernatant was used for protein quantification and immunoblotting/kinase assays as described below. Four hundred mg of protein were incubated with 2 ml of CDK4, CDK2, CDK1 (Gibco BRL, Rockville, MD, USA) and CDK6 antisera (manuscript in preparation) for 1 h in a shaker at 4°C followed by the addition of 20 ml Gammabind G Sepharose (Pharmacia, Piscataway, NJ) suspension (50%). After a further incubation for 1 h at 4°C, the immunocomplex was centrifugated at 800 ***g*** for 1 min, washed 3 times with lysing buffer containing 0.5 M NaCl, and once with kinase buffer (50 mM Hepes, pH 7.5, 10 mM MgCl2, 5 mM MnCl, 1 mM DTT, 10 mM β-glycerophosphate, 2.5 mM EGTA, 0.5 mM NaF, 0.4 mM $NaVO_4$). The kinase reaction was started by the addition of 30 ml kinase buffer containing 5 mM ATP 1 mCi of 32P-ATP and 1 mg of GST-Rb (histone H1 for CDK2 and CDK1 from Boehringher Mannheim). The reaction mixture was incubated for 30 min at 30°C with constant mixing, and stopped by adding 6 ml of 6× SDS-sample buffer followed by heating for 5 min at 95°C. After a quick centrifugation, proteins (30 ml) were separated by SDS-PAGE, using a 12% acrylamide gel. The gels were dried and radio-labelled substrate was quantified in a Storm phosphorimager (Molecular Dynamics, Sunnyvalley, CA, USA).

### Cell number

Briefly, 10 000–15 000 cells were plated in 24-well plates in 1 ml of medium. The next day, cells were treated with depsipeptide. After 4 days, cells were trypsinized and each condition was counted in triplicate on a Coulter Z1 cell counter (Hialeah, FL, USA).

### DNA synthesis

DNA synthesis was monitored by $^3$H-thymidine incorporation. In brief, 2000 cells were plated in 96-well flat bottom plates or 15 000 cells were plated in 24-well plates. The next day, cells were treated with drugs and incubated for 16 hours. Then, cells were incubated with 1 μCi [methyl$^3$H]-thymidine (Amersham) for an additional 4 hours and then acid-insoluble radioactivity was determined.

### RESULTS

### p53-independent cell cycle arrest in a panel of cell lines

To confirm that depsipeptide, FR901228, causes G1 cell cycle arrest in human cancer cells as previously reported for H-Ras transformed NIH 3T3 cells (Ueda et al, 1994c), we analysed the

 © 2000 Cancer Research Campaign

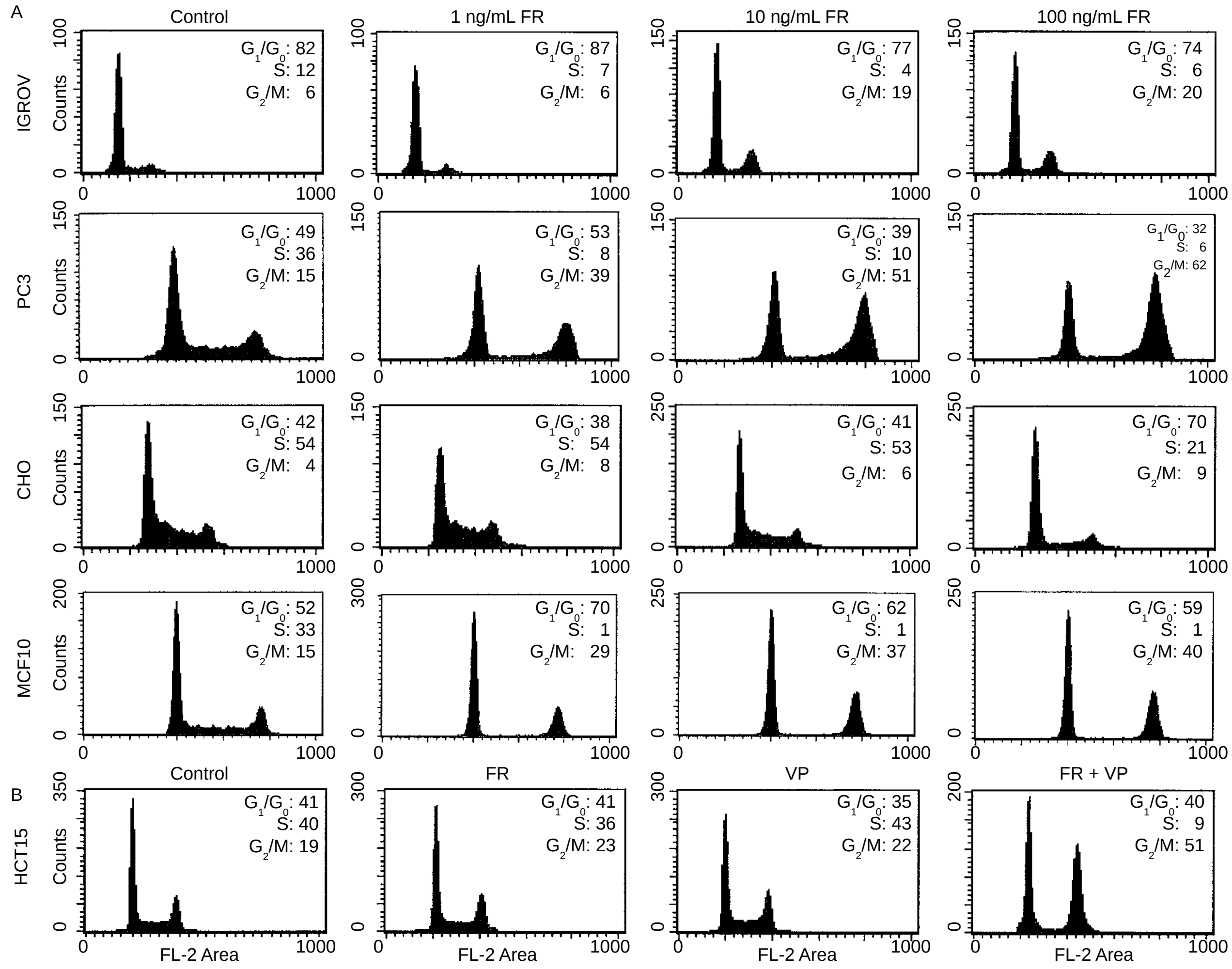

**Figure 1** Effects of FR90128 on cell cycle and G1 and G2/M cell cycle arrest. (**A**) IGROV, PC3, CHO, MCF10 cells were treated with 1 ng/ml, 10 ng/ml, or 100 ng/ml of FR901228 (FR) or left untreated (control) for 24 h, and cell cycle analysis was performed as described in Methods. The experiments were repeated three times. (**B**) HCT-15 cells were treated with 10 ng/ml FR901228 (FR), 10 ng/ml verapamil (VP), or their combination (FR+VP) for 24 h and cell cycle analysis was performed as described in Methods effect of FR901228 on the cell cycle distribution of several cell lines from the NCI anticancer drug screen. Figure 1 shows representative cell cycle histograms at three different concentrations of FR901228. In addition to a G1 cell cycle arrest, a G2 cell cycle arrest can be seen. The ability of FR901228 to cause a G1 cell cycle arrest in the p53-null PC3 cell line suggests a p53-independent effect. Although the number of PC3 cells in G1 was unchanged after FR exposure, the disappearance of S cells indicates a G1 arrest. An unchanged number of cells in G1, despite the disappearance of the S phase cells, can be explained if cells neither enter G1 (due to G2/M arrest) nor exit G1 (G1 arrest). In the absence of G1 arrest, cells would accumulate exclusively in G2/M phase (see for example the p21–/– cells in Figure 7).

Although FR901228 increased both G1 and G2 fractions, higher doses (100 ng/ml) caused a predominance of the G2 phase arrest. CHO cells were relatively resistant to FR901228 whereas minimally transformed MCF10 cells were the most sensitive. HCT-15 cells were also resistant to FR901228. However, HCT-15 cells are known to express significant levels of the multidrug transporter Pgp, and since FR901228 was previously shown to be a substrate for efflux by Pgp (Lee et al, 1994), 10 ng/ml verapamil was added in combination with FR901228. Subsequently, a distinct G1 and G2 arrest pattern emerged (Figure 1 B).

### FR901228 arrested cells in G1 before the restriction point of DNA synthesis

To further define the G1 cell cycle arrest caused by FR901228, the immortalized EGF-dependent breast cell line MCF-10A was used (Figure 2). Synchronization of these cells in G0/G1 was achieved with 24 h growth factor or serum deprivation (Figure 2A, Starvation). Following release into serum-containing medium these cells redistributed normally in the cell cycle within 18 hours of release (Release). However, when these synchronized cells were released into serum-containing medium with 10 ng/ml FR901228, they remained in G0/G1 (Release + FR) in contrast to the G1 and G2/M arrest that occurs in exponentially growing cells following FR901228 treatment (FR). Aphidicolin was used to further define the point at which G1 arrest occurs. Aphidicolin reversibly inhibits DNA polymerase causing a cell cycle arrest at the G1/S border after the restriction point (Figure 2B). With synchronization in G1/S achieved using aphidicolin, cells released

© 2000 Cancer Research Campaign

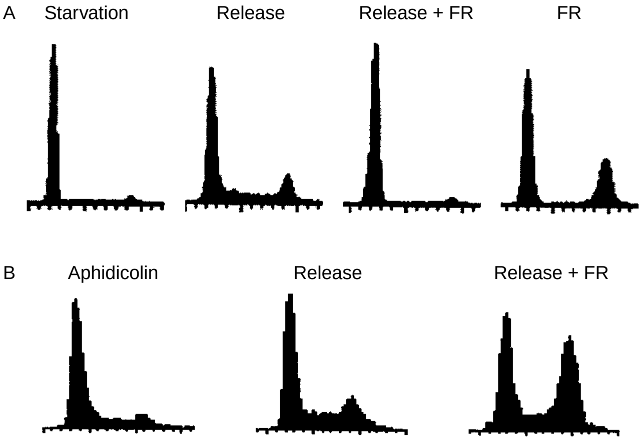

**Figure 2** Effects of FR901228 on cell cycle distribution in MCF10 cells synchronized by serum starvation (**A**) or aphidicolin (**B**). (**A**) Effects of FR901228 on G1 progression in G0-synchronized MCF10 cells. MCF10 cells can be arrested in G0/G1 due to 24 h serum starvation (Starvation). Following a release into serum-containing medium (Release), cells resume S phase. Cells released into serum-containing medium with 10 ng/ml FR (Release + FR) maintain the cell cycle arrest in G1. Unsynchronized MCF10 cells treated with 10 ng/ml FR for 16 h (FR) show a G1 and a G2/M arrest. The experiments were repeated 3 times. (**B**) Effects of the Aphidicolin-pretreatment on the growth arrest caused by FR901228. MCF10 cells were synchronized by treatment with 3 μM aphidicolin for 24 h (Aphidicolin). After synchronization with aphidicolin, cells were released into aphidicolin-free medium for 18 h (Release), or into aphidicolin-free medium containing 10 ng/ml FR for 18 h (Release+FR)

into serum-containing, aphidicolin-free medium redistributed like exponentially growing cells within the cell cycle at 18 hours (Figure 2, Release). Cells released from aphidicolin-containing medium into aphidicolin-free medium containing 10 ng/ml FR901228, cycled through S phase and were arrested in G2/M (Release + FR). This suggests that FR901228 causes cell cycle arrest in early G1, before the G1/S restriction point, and also confirms the G2/M arrest.

### FR901228 induced Rb dephosphorylation

The retinoblastoma protein (Rb) is a central regulatory protein in the G1-to-S transition (Weinberg, 1995). Inactivation of Rb through hyperphosphorylation by cyclin dependent kinase (CDK) complexes such as cyclin D/CDK4 and cyclin E/CDK2 causes the release of the E2F transcription factor which in turn controls the transcription of genes required for the G1-to-S transition (DeGregori et al, 1995; Nevins, 1998; Sherr, 1999). Conversely, hypophosphorylated forms of Rb bind to the E2F family of transcription factors preventing their action and, complexed to E2F, act as active transcriptional repressors preventing cell cycle progression (Zhang et al, 1999). The immunoblot for Rb in Figure 3 shows a time course for MCF-10A and PC-3 cells treated with FR901228. Several bands can be seen representing Rb protein, with the higher molecular weight bands representing hyper-phosphorylated forms of Rb and the lower molecular weight bands representing hypophosphorylated forms of Rb. Within 6–8 hours of treatment with 10 ng/ml of FR901228, the hypophosphorylated forms of Rb predominate in both MCF-10A cells and PC-3 cells. After 12 hours of treatment, Rb is almost completely in the hypophosphorylated form in both cell lines. In addition to pRb dephosphorylation, the overall level of pRb appears to be

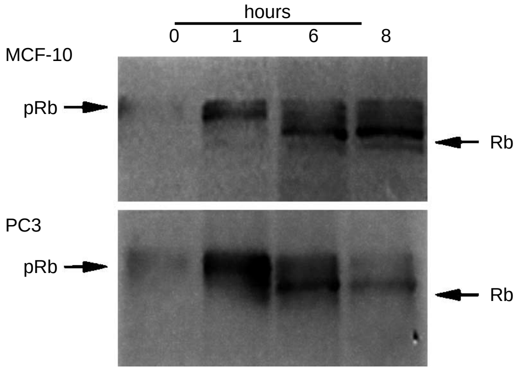

**Figure 3** Effects of FR901228 on Rb phosphorylation. MCF10A and PC3 cells were treated with 10 ng/ml FR901228 (FR) for the indicated time and immunoblot for Rb was performed as described in Methods. The experiments were repeated 3 times

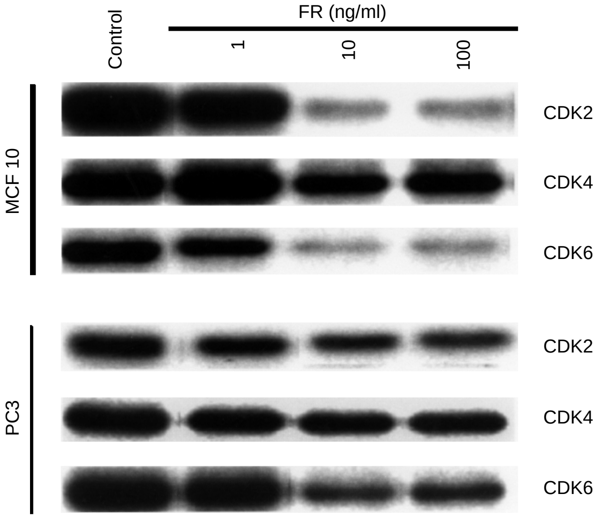

**Figure 4** Effects of FR901228 on CDK activities in vivo. MCF10A cells and PC3 cells were treated with 1 ng/ml, 10 ng/ml, and 100 ng/ml FR for 14 h and measurement of CDK kinase activity was performed as described in the Methods. The representative results of three experiments are shown upregulated. As suggested by Juan et al, the increase in quantity of Rb may act as an additional factor arresting cells in G1, as observed during cell differentiation (Juan et al, 1998).

### FR901228 inhibits CDK by an indirect mechanism

Next, in vitro kinase assays were performed to examine the effect of FR901228 on CDK2 and CDK4 activity. CDK2 and CDK4 were immunoprecipitated from exponentially growing PC3 cells and kinase assays performed in the presence or absence of 1, 10, and 100 ng/ml of FR901228. No direct CDK2 or CDK4 inhibition by FR901228 was noted (data not shown). Figure 4 shows the results of a similar experiment performed in living cells. PC3 cells were treated with FR901228, and CDK were then precipitated from extracts of these cells, and in vitro kinase assays performed. Inhibition of CDK2, 4 and 6 activity was noted after treatment of cells with FR901228 (Figure 4). The in vivo inhibition in the absence of in vitro inhibition suggests that depsipeptide is acting on pathways upstream of CDK.

 *© 2000 Cancer Research Campaign*

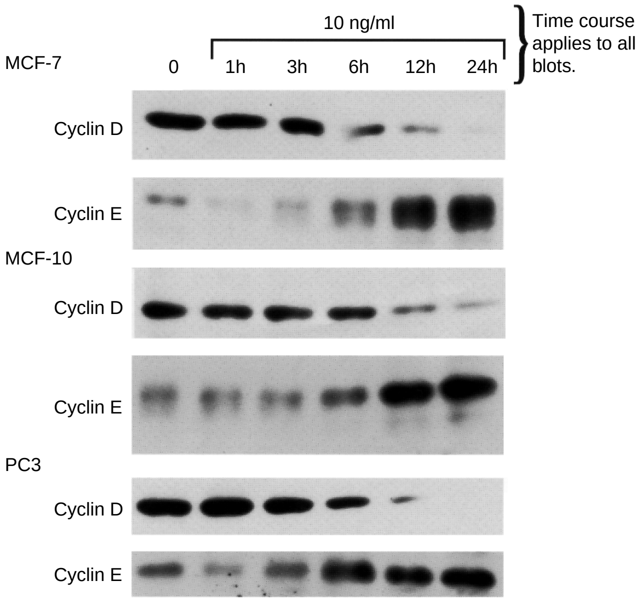

**Figure 5** Effects of FR901228 on cyclin D and cyclin E. MCF-7, MCF10A, and PC3 cells were treated with 10 ng/ml FR for the indicated times and immunoblot for cyclin D1 and cyclin E were performed as described in Methods

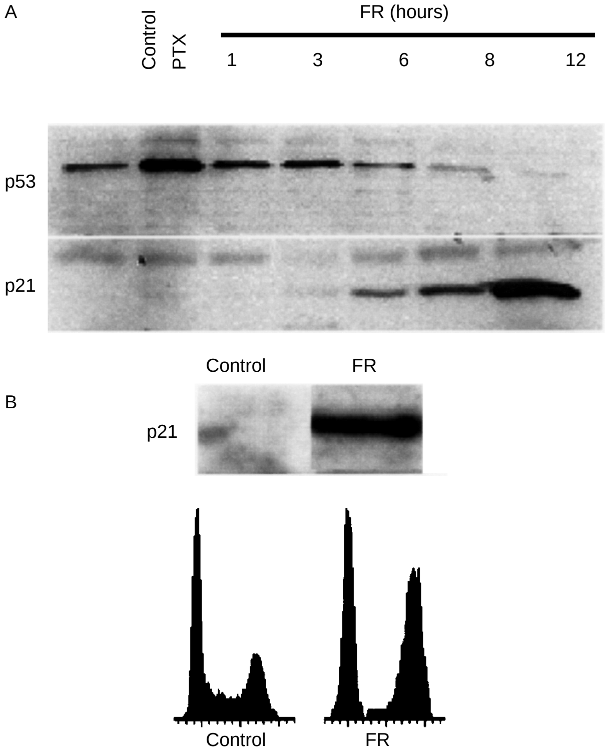

**Figure 6** Effects of FR901228 on the p53-independent induction of p21 and growth arrest. (**A**) MCF10A (wt p53) cells were treated with 10 ng/ml FR for the indicated times or with 100 ng/ml paclitaxel (PTX) and immunoblot analysis for p53 and p21 was performed as described in the Methods. (**B**) PC3 (p53-null) cells were treated with 10 ng/ml FR for 12 h and immunoblot for p21 was performed as described in the Methods. Cell cycle distribution of PC3 cells by 16 h of treatment is shown below

### FR901228 down-regulated cyclin D1 and up-regulated p21

Because CDKs are activated by cyclins and inhibited by CDK inhibitors such as p21, we examined the effect of FR901228 on cyclin D1 and p21. Cyclin D1 was found to be decreased by 6 h and absent after 12 h of treatment with 10 ng/ml of FR901228 in MCF-7, MCF-10A and PC3 cells (Figure 5). In contrast, an increase in cyclin E was observed after treatment with FR901228. An increase in cyclin E should result in rescue of the CDK activity unless an inhibitor of the complex is present.

$p21^{WAF1/CIP1}$ is capable of inhibiting both G1- and G2-cyclin/CDK complexes, thus causing both a G1 and G2 cell cycle arrest. In addition to wt p53 (El-Deiry et al, 1993), numerous compounds induce p21 independently of p53. Induction of p21 with down-regulation of wt p53 is noted in MCF-10A (Figure 6). Also, p21 was induced by 12 h in PC3 cells associated with both G1 and G2/M arrest (Figure 6B). Thus, FR901228 causes p21 WAF1 induction independently of p53. This result is compatible with the p53 independence of FR901228 action noted in Figure 1.

### p21 was required for G1 arrest but not for cytotoxicity

Since p21 was induced by FR901228, we next investigated the significance of that induction in FR901228-mediated growth arrest. We took advantage of the availability of two clones of the HCT116 colon cancer cell line, designated as S4 and S14, which were engineered to lack the p21 gene. These two p21-knockout clones differ from parental HCT116 cells only by the missing p21 genes (Waldman et al, 1995, 1996; Bunz et al, 1998). As expected, p21 protein was undetected in p21–/– clones (Figure 7 A and data not shown) but was induced by FR901228 in HCT116 cells (Figure 7 A). Cyclin D1 was downregulated in both parental HCT116 and p21–/– cells following FR901228 treatment (Figure 7 A). Absence of p21 in cells corresponded to failure of FR901228 to cause G1 arrest and to inhibit $^{3}$H-thymidine incorporation indicating ongoing DNA synthesis in cells lacking the p21 genes (Figure 7 B, C). The cell cycle distribution was similar in untreated clones and parental cells (Figure 7 B). However, following 24 hours of exposure to 10 ng/ml FR901228, HCT116 cells were arrested in both G1 and G2/M phases of the cell cycle. In contrast, p21-deficient S4 and S14 clones were arrested exclusively in G2/M phase but not in G1 phase. This lack of G1 arrest confirmed the ability of p21-deficient clones to continue DNA synthesis ($^{3}$H-thymidine incorporation) following FR901228 treatment (Figure 7 C).

Further, FR901228 was not only toxic to p21-deficient cells but was more toxic than to parental cells. Thus, following 2 days of treatment with 10 ng/ml of FR901228 approximately 16% of parental HCT116 cells survived whereas only 3–4% of S4 cells could be detected (Figure 7D). Comparable cytotoxicity in HCT116 cells required an additional 24–48 h of exposure. Cytoprotective role of p21 induction and G1 arrest was previously demonstrated in p53-mediated apoptosis in HCT116 cells (Polyak et al, 1996). Our result suggests that the G2/M arrest may be more effective in achieving cell death than the G1 arrest following FR901228 treatment.

We next examined the effects of p21 on cell cycle distribution in HCT116 following infection of these cells with p21-expressing adenovirus. Following p21 overexpression, the number of cells in G1 phase was increased from 52% to 75%, the number of cells in

© 2000 Cancer Research Campaign

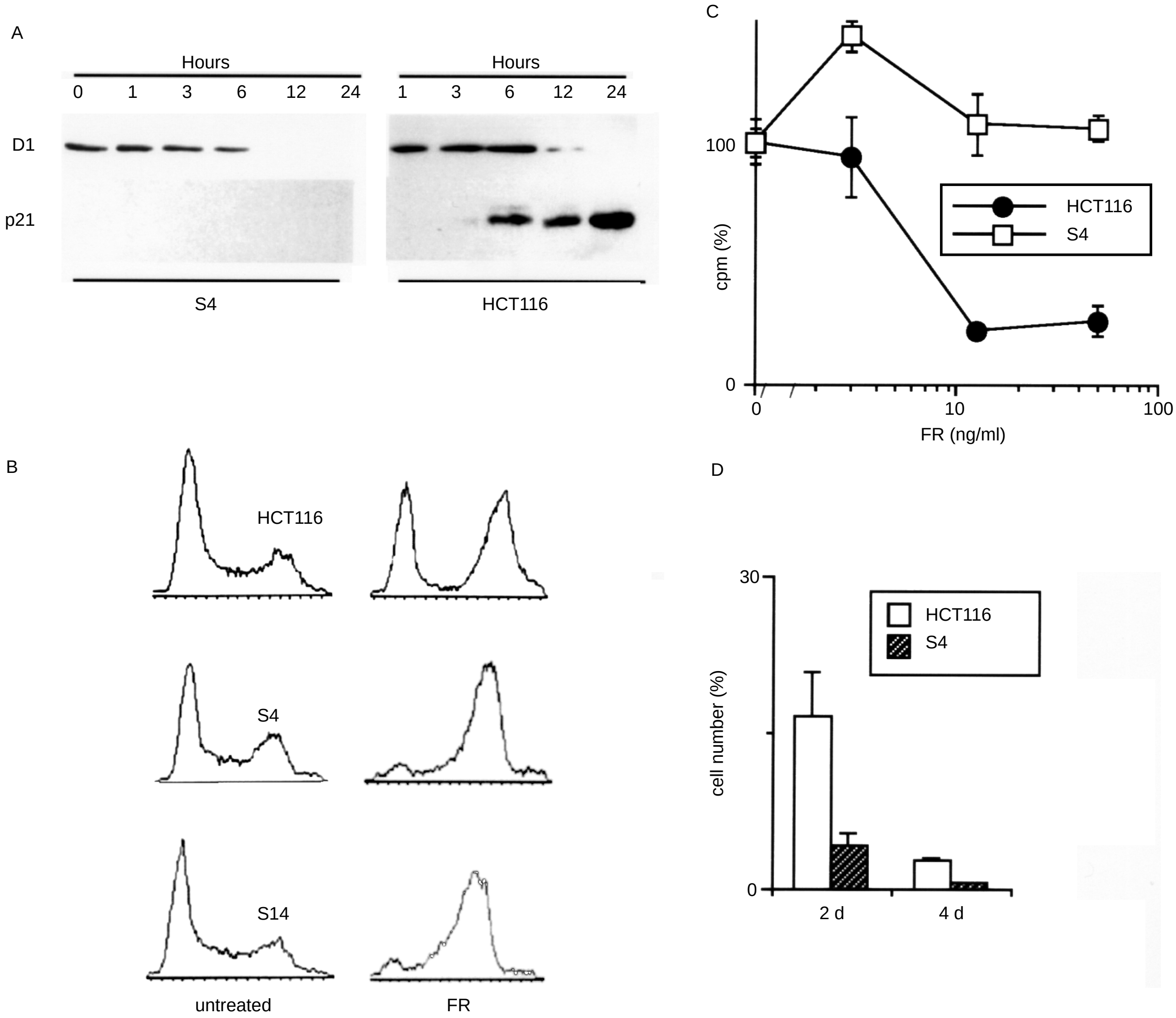

**Figure 7** The role of p21 in the G1 arrest and cytotoxicity caused by FR901228. (**A**) Immunoblot analysis of cyclin D1 and p21 following FR treatment. S4, a p21-deficient clone, and parental HCT116 cells were treated with 10 ng/ml FR for the indicated times and immunoblots for cyclin D1 and p21 were performed as described in the Methods. (**B**) Cell cycle distribution. Parental HCT116 cells and S4 and S14 clones lacking p21 were treated with 10 ng/ml FR901228 (FR) or left untreated (control) for 24 h, and cell cycle analysis was performed as described in the Methods. (**C**) Parental HCT116 cells (closed circles) and S4 cells lacking p21 (open squares) were treated with indicated concentrations of FR (ng/ml) for 16 h, and 3H-thymidine incorporation was performed in triplicate as described in the Methods. Results represent percent of the control value in untreated cells (mean ± SD). (**D**) Parental HCT116 cells (open bars) and S4 cells lacking p21 (closed bars) were treated with 10 ng/ml FR for the indicated time, and the number of live cells was counted in triplicate as described in the Methods. Results represent percent of value of untreated cells (mean ± SD).

S phase was decreased from 30% to 10% and the number of G2/M cells was unchanged (18% and 16%, respectively). Therefore, overexpression of p21 alone (without FR901228) exerts growth arrest in the G1 phase in HCT116 cells, whereas the absence of endogenous p21 in p21–/– cells precludes G1 growth arrest.

### FR901228 prevented MAPK activation but not tyrosine phosphorylation

Growth factor (GF) signalling is required for the passage of normal cells from G0/G1 through the restriction point and into the cell cycle (Pardee, 1974). These signalling pathways represent an upstream regulatory component of CDK activity and Rb phosphorylation state. GF activates several signal transduction pathways including the ERK2/MAPK pathway. MCF-10A cells were used to examine the effect of FR901228 on signal transduction through MAPK. Figure 8A shows a mobility shift assay for activation of ERK-2 in MCF-10A cells that were serum starved for 24 hours, exposed to 0, 1, 10, or 100 ng/ml of FR901228 for 16 hours; and then stimulated with 10 ng/ml EGF for 5 minutes before harvesting. The first lane shows the control, which received no EGF stimulation or treatment with FR901228, and as expected shows no shift in the mobility of the ERK-2 band. The next lane demonstrates the MAPK phosphorylation after EGF stimulation, in the absence of FR901228. The subsequent three lanes show a dose-dependent decrease in the overall quantity of ERK-2 activation relative to the quantity that remains inactive.

Early events in the EGF signal transduction pathway involve sequential tyrosine phosphorylation of proteins (Hunter, 1997). The effect of FR901228 on early tyrosine phosphorylation events was examined to determine whether FR901228 disrupts the EGF/MAPK pathway through direct inhibition of tyrosine

 © 2000 Cancer Research Campaign

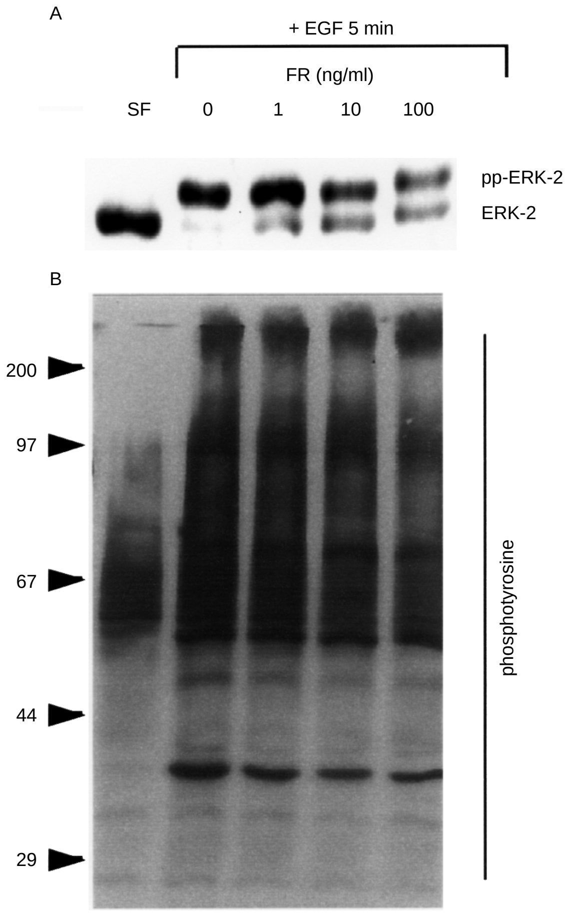

**Figure 8** Effects of FR901228 on the MAPK pathway and tyrosine kinase activation. MCF 10 cells were synchronized in serum-free medium (SF), and then stimulated with 10 ng/ml EGF for five minutes. As shown, cells were pretreated with the indicated doses of FR for 16 hours prior to lysis. Cells were lysed and an immunoblot for ERK2 (**A**) and total phosphotyrosine (**B**) was performed as described in the Methods.

phosphorylation. An immunoblot of cell lysates prepared as described above was probed for phosphotyrosine using an antiphosphotyrosine antibody (Figure 8 B). The first lane shows a low level of tyrosine phosphorylation in lysates from serum deprived cells. Lysates from cells stimulated with EGF for 5 minutes after treatment with 0, 1, 10 and 100 ng/ml of FR901228 show no differences in overall tyrosine phosphorylation suggesting that depsipeptide does not act as a general tyrosine kinase inhibitor, and also that FR901228 does not disrupt early signal transduction events regulated by tyrosine phosphorylation.

### DISCUSSION

We have characterized an aspect of the mechanism of action of FR901228 by defining its effect on pathways that regulate G1-to-S transition. Cell cycle studies using synchronized populations of MCF-10A cells suggest that FR901228 blocks cell cycle progression before the G1 phase restriction point. Consistently, Rb protein becomes hypophosphorylated after 6 hours of treatment with FR901228. This hypophosphorylation can be explained by a decrease in CDK activity seen after cells are exposed to FR901228. This drop in kinase activity seems to result from a decrease in cyclin D1 protein, a CDK activator, and an increase in p21 protein, a CDK inhibitor, after exposure to the drug. These changes were accompanied by a decrease in cyclin D1 mRNA and increase in p21 mRNA (data not shown). This identified FR901228 as a compound capable of inducing p21 independently of p53. Furthermore, cells without p21 expression did not undergo FR901228-mediated G1 arrest. This provides evidence for p21 induction as the mediator for the G1 arrest caused by FR901228.

The D family of cyclins act as growth factor sensors and are induced as a response to growth factor stimulation (Sherr, 1999). Our findings show that FR901228 disrupts signal transduction through the ERK2/MAPK pathway. Overall cellular tyrosine phosphorylation in response to EGF stimulation remains intact suggesting that immediate events at the EGF pathway are not disrupted. We conclude that the G1 cell cycle arrest caused by FR901228 can be explained by disruption of signal transduction leading to down-regulation of cyclin D1. The only known biochemical activity of FR901228 is inhibition of histone deacetylase (Nakajima et al, 1998). Our findings are consistent with results that have been observed with other agents known to inhibit histone deacetylases. Oxamflatin, another histone deacetylase inhibitor, increased transcriptional expression of cyclin E and CDK inhibitor p21 and decreased expression of cyclin D1 (Kim et al, 1999). Butyrate is another histone deacetylase inhibitor which in millimolar concentrations causes G1 arrest before the restriction point of G1/S progression (Campisi et al, 1982; Vaziri et al, 1998). Importantly, FR901228 caused G1 arrest and cytotoxicity at nanomolar concentrations, thus showing at least 100 000-fold higher activity than butyrate.

In normal cells, induction of p21, a CDK inhibitor, opposes the effects of cyclins D and E on CDK which enables it to interrupt the cell cycle (Sherr, 1999). However, cyclin levels are not normally reduced by p21 induction (Chen et al, 1995). While it has been reported that growth arrest by butyrate was mediated by p21 in HCT116 cells (Archer et al, 1998), another group found that p21 induction is dispensable for the G1 arrest by butyrate in mouse fibroblasts (Vaziri et al, 1998). In contrast to studies in fibroblasts (Vaziri et al, 1998) and MOLT-4 cells (Gong et al, 1994) that demonstrated a decrease in cyclin E following butyrate treatment, in our study, FR901228 up-regulated cyclin E in human cancer cell lines. While our manuscript was under review, it was reported that Rb forms a repressor containing histone deacetylase which inhibits transcription of genes for cyclins E (Zhang et al, 2000).

If cells are arrested by serum starvation in G0, cyclin D levels are diminished, and Rb, the substrate of the cyclin D-activated CDK, is hypophosphorylated. FR901228 can maintain this status and prevent induction of cyclin D1 and Rb phosphorylation, causing G1 arrest. It has been shown that, while E2F induces cyclin E (Botz et al, 1996), dephosphorylated Rb complexed with E2F actively blocks cyclin E expression, thus preventing the next step of cell cycle progression (Zhang et al, 1999).

Thus, the apparent contradictory down-regulation of cyclin D1 and up-regulation of cyclin E may be explained by the inhibitory effects of FR901228 on histone deacetylase. Dephosphorylated Rb complexes with E2F, and recruiting a histone deacetylase, exerts active transcriptional repression (Brehm et al, 1998; Luo et al, 1998; Magnaghi-Jaulin et al, 1998; Zhang et al, 1999). Therefore, histone deacetylase may be responsible for repression of cyclin E during natural G1 arrest. FR901228, by inhibiting histone deacetylase may prevent cyclin E down-regulation. Simultaneously, inhibition of

© 2000 Cancer Research Campaign 

histone deacetylase transcriptionally induces p21 (Sowa et al, 1997), which we observed following FR901228 treatment. It is noteworthy that while cyclins D1 and E, and the CDK inhibitor p21 are commonly induced in parallel (Blagosklonny, 1999), FR901228 treatment resulted in a unique combination: increased p21 and cyclin E accompany decreased cyclin D1.

This induction of cyclin E, despite cyclin D down-regulation and initial Rb dephosphorylation, could move cells into S phase. However, p21 is also induced at the time of cyclin E induction, and it has been shown that p21 can inhibit cyclin E-driven initiation of S phase (Stewart et al, 1999). Therefore, increased p21 counter-balances increased cyclin E following FR901228 treatment. Sustained G1 arrest, which is initially triggered by downregulation of cyclin D1, thus depends on p21 expression. Cells lacking p21 (p21–/– cells) enter S phase and arrest in G2/M. p21 induction was dispensable for G1 arrest by butyrate in MEF cells in which butyrate down-regulated cyclin E (Vaziri et al, 1998). If both cyclins D and E are down-regulated, induction of p21 becomes dispensable for growth arrest. Thus, the difference in cyclin E regulation may determine the requirement for p21 induction by FR901228. In contrast, neither the G2/M arrest nor cytotoxicity of depsipeptide depends on p21. Therefore, this promising experimental therapeutic may have several targets that determine its unique cytotoxic profile. Further studies will define the association between the G1 cell cycle arrest caused by FR901228 and its ability to induce G2/M-associated cytotoxic effects.

### REFERENCES

Aktas H, Cai H and Cooper GM (1997) Ras links growth factor signaling to the cell cycle machinery via regulation of cyclin D1 and the Cdk inhibitor p27KIP1. *Mol Cell Biol* **17**: 3850–3857

Archer SY, Meng S, Shei A and Hodin RA (1998) p21WAF1 is required for butyrate-mediated growth inhibition of human colon cancer cells. *Proc Natl Acad Sci USA* **95**: 6791–6796

Blagosklonny MV (1999) A node between proliferation, apoptosis, and growth arrest. *BioEssays* **21**: 704–709

Blagosklonny MV, Somasundaram K, Wu GS and El-Deiry WS (1997) Wild-type p53 is not sufficient for serum starvation-induced apoptosis in cancer cells but accelerates apoptosis in sensitive cells. *Int J Oncol* **11**: 1165–1170

Botz J, Zerfass-Thome K, Spitkovsky D, Delius H, Vogt B, Eilers M, Hatzigeorgiou A and Jansen-Durr P (1996) Cell cycle regulation of the murine cyclin E gene depends on an E2F binding site in the promoter. *Mol Cell Biol* **16**: 3401–3409

Brehm A, Miska EA, McCance DJ, Reid JL, Bannister AJ and Kouzarides T (1998) Retinoblastoma protein recruits histone deacetylase to repress transcription. *Nature* **391**: 597–601

Bunz F, Dutriaux A, Lengauer C, Waldman T, Zhou S, Brown JP, Sedivy JM, Kinzler KW and Vogelstein B (1998) Requirement for p53 and p21 to sustain G2 arrest after DNA damage. *Science* **282**: 1497–1501

Campisi J, Medrano EE, Morreo G and Pardee A (1982) Restriction point control of cell growth by a labile protein: evidence for increased stability in transformed cells. *Proc Natl Acad Sci USA* **79**: 436–440

Candido EPM, Reeves R and Davie JR (1978) Sodium butyrate inhibits histone deacetylation in cultured cells. *Cell* **14**: 105–113

Chen X, Bargonetti J and Prives C (1995) P53, through p21 (WAF1/CIP1), induces cyclin D1 synthesis. *Cancer Res* **55**: 4257–4263

DeGregori J, Leone G, Ohtani K, Miron A and Nevins J (1995) E2F-1 accumulation bypasses a G1 arrest resulting from the inhibition of G1 cyclin-dependent kinase activity. *Genes Dev* **9**: 2873–2887

El-Deiry WS, Tokino T, Veculescu VE, Levy DB, Parsons R, Trent JM, Lin D, Mercer WE, Kinzler KW and Vogelstein B (1993) WAF1, a potential mediator of p53 tumor suppression. *Cell* **75**: 817–825

Gong JP, Traganos F and Darzynkiewicz Z (1994) Use of the cyclin E restriction point to map cell arrest in G(1)-induced by N-butyrate, cycloheximide, staurosporine, lovastatin, mimosine and quercetin. *Int J Oncol* **4**: 803–808

Grignani F, De Matteis S, Nervi C, Tomassoni L, Gelmetti V, Cioce M, Fanelli M, Ruthardt M, Ferrara FF, Zamir I, Seiser C, Grignani F, Lazar MA, Minucci S and Pelicci PG (1998) Fusion proteins of the retinoic acid receptor-alpha recruit histone deacetylase in promyelocytic leukemia. *Nature* **391**: 815–818

Hunter T (1997) Oncoprotein networks. *Cell* **88**: 333–346

Johnson DG, Schwarz JK, Cress WD and Nevins JR (1993) Expression of transcription factor E2F1 induces quiescent cells to enter S-phase. *Nature* **365**: 349–352

Juan G, Li X and Darzynkiewicz Z (1998) Phosphorylation of retinoblastoma protein assayed in individual HL-60 cells during their proliferation and differentiation. *Exp Cell Res* **244**: 83–92

Kim YB, Lee KH, Sugita K, Yoshida M and Horinouchi S (1999) Oxamflatin is a novel antitumor compound that inhibits mammalian histone deacetylase. *Oncogene* **18**: 2461–2470

Kosugi H, Towatari M, Hatano S, Kitamura K, Kiyoi H, Kinoshita T, Tanimoto M, Murate T, Kawashima K, Saito H and Naoe T (1999) Histone deacetylase inhibitors are the potent inducer/enhancer of differentiation in acute myeloid leukemia: a new approach to anti-leukemia therapy. *Leukemia* **13**: 1316–1324

Lee JS, Paull K, Alvarez M, Hose C, Monks A, Grever M, Fojo AT and Bates SE (1994) Rhodamine efflux patterns predict P-glycoprotein substrates in the National Cancer Institute drug screen. *Mol Pharmacol* **46**: 627–638

Lin RJ, Nagy L, Inoue S, Shao W, Miller WH and Evans RM (1998) Role of the histone deacetylase complex in acute promyelocytic leukemia. *Nature* **391**: 811–814

Luo RX, Postigo AA and Dean DC (1998) Rb interacts with histone deacetylase to repress transcription. *Cell* **92**: 463–473

Magnaghi-Jaulin L, Groisman R, Naguibneva I, Robin P, Lorain S, Le Villain JP, Troalen F, Trouche D and Harel-Bellan A (1998) Retinoblastoma protein repress transcription by recruiting a histone deacetylase. *Nature* **391**: 601–605

Nakajima H, Kim YB, Terano H, Yoshida M and Horinouchi S (1998) FR901228, a potent antitumor antibiotic, is a novel histone deacetylase inhibitor. *Exp Cell Res* **241**: 126–133

Nevins JR (1998) Toward an understanding of the functional complexity of the E2F and retinoblastoma families. *Cell Growth Diff* **9**: 585–593

Oswald F, Lovec H, Moroy T and Lipp M (1994) E2F-dependent regulation of human MYC: trans-activation by cyclins D1 and A overrides tumour suppressor protein function. *Oncogene* **9**: 2029–2036

Pardee AB (1974) A restriction point for control of normal animal cell proliferation. *Proc Natl Acad Sci USA* **71**: 1286–1290

Polyak K, Waldman T, He T-C, Kinzler KW and Vogelstein B (1996) Genetic determinants of p53-induced apoptosis and growth arrest. *Genes Dev* **10**: 1945–1952

Rajgolikar G, Chan KK and Wang HC (1998) Effects of a novel antitumor depsipeptide, FR901228, on human breast cancer cells. *Breast Cancer Res Treat* **51**: 29–38

Richon VM, Emiliani S, Verdin E, Webb Y, Breslow R, Rifkind RA and Marks PA (1998) A class of hybrid polar inducers of transformed cell differentiation inhibits histone deacetylases. *Proc Natl Acad Sci USA* **95**: 3003–3007

Sherr CJ (1999) Cancer cell cycles. *Science* **274**: 1672–1677

Soule HD, Maloney TM, Wolman SR, Peterson WD, Brenz R, McGrath CM, Russo J, Pauley RJ, Jones RF and Brooks SC (1990) Isolation and characterization of a spontaneously immortalized human breast epithelial cell line, MCF-10. *Cancer Res* **50**: 6075–6086

Sowa Y, Orita T, Minamikawa S, Nakano K, Mizuno T, Nomura H and Sakai T (1997) Histone deacetylase inhibitor activates the WAF1/Cip1 gene promoter through the SP1 sites. *Biochem Biophys Res Commun* **241**: 142–150

Stewart ZA, Leach SD and Pietenpol JA (1999) p21WAF1/Cip1 inhibition of cyclin E/Cdk2 activity prevents endoreduplication after mitotic spindle disruption. *Mol Cell Biol* **19**: 205–215

Ueda H, Manda T, Matsumoto S, Mukumoto S, Nishigaki F, Kawamura I and Shimomura K (1994a) FR901228, a novel antitumor bicyclic depsipeptide produced by *Chromobacterium violaceum* No. 968. Antitumor activities on experimental tumors in mice. *J Antibiot* **47**: 315–323

Ueda H, Nakajima H, Hori Y, Fujita T, Nishimura M, Goto T and Okuhara M (1994b) FR901228, a novel antitumor bicyclic depsipeptide produced by *Chromobacterium violaceum* No. 968. I. Taxonomy, fermentation, isolation, physico-chemical and biological properties, and antitumor activity. *J Antibiot* **47**: 301–310

Ueda H, Nakajima H, Hori Y, Goto T, Okuhara M (1994c) Action of FR901228, a novel antitumor bicyclic depsipeptide produced by *Chromobacterium violaceum* no. 968, on Ha-ras transformed NIH3T3 cells. *Biosci Biotechnol Biochem* **58**: 1579–1583

Vaziri C, Stice L and Faller DV (1998) Butyrate-induced G1 arrest results from p21-independent disruption of retinoblastoma protein-mediated signals. *Cell Growth Diff* **9**: 465–474

Waldman T, Kinzler KW and Vogelstein B (1995) p21 is necessary for the p53-mediated G1 arrest in human cancer cells. *Cancer Res* **55**: 5187–5190

© 2000 Cancer Research Campaign

Waldman T, Lengauer C, Kinzler KW and Vogelstein B (1996) Uncoupling of S phase and mitosis induced by anticancer agents in cells lacking p21. *Nature* **381**: 643–644

Wang R, Brunner T, Zhang L and Shi Y (1998) Fungal metabolite FR901228 inhibits c-Myc and Fas ligand expression. *Oncogene* **17**: 1503–1508

Weinberg RA (1995) The retinoblastoma protein and cell cycle control. *Cell* **81**: 323–330

Wosikowski K, Regis JT, Robey RW, Alvarez M, Buters JTM, Gudas JM and Bates SE (1996) Normal p53 status and function despite the development of drug resistance in human breast cancer cells. *Cell Growth Differentiation* **6**: 1395–1403

Zhang HS, Postigo AA and Dean DC (1999) Active transcriptional repression by the Rb-E2F complex mediates G1 arrest triggered by p16INK4a, TGFbeta, and contact inhibition. *Cell* **97**: 53–61

Zhang HS, Gavin M, Dahiya A, Postigo AA, Ma D, Luo RX, Harbour JW and Dean DC. (2000) Exit from G1 and S phase of the cell cycle is regulated by repressor complexes containing HDAC-Rb-hSWI/SNF and Rb-hSWI/SNF. *Cell* **101**: 79–89

Zou X, Rudchenko S, Wong K and Calame K (1997) Induction of c-myc transcription by the v-Abl tyrosine kinase requires Ras, Raf1 and cyclin-dependent kinases. *Genes & Dev* **11**: 654–662

*© 2000 Cancer Research Campaign*
